# Outlook on RNAi-Based Strategies for Controlling *Culicoides* Biting Midges

**DOI:** 10.3390/pathogens12101251

**Published:** 2023-10-17

**Authors:** Cameron J. Osborne, Lee W. Cohnstaedt, Kristopher S. Silver

**Affiliations:** 1 Department of Entomology, College of Agriculture, Kansas State University, Manhattan, KS 66506, USA; cjosborne@ksu.edu; 2 Foreign Arthropod-Borne Animal Diseases Research Unit, National Bio- and Agro-Defense Facility, Agricultural Research Service, United Stated Department of Agriculture, Manhattan, KS 66502, USA

**Keywords:** RNA interference, larvicide, bio-rational insecticide, microbial expression, nanoparticles

## Abstract

*Culicoides* are small biting midges with the capacity to transmit important livestock pathogens around much of the world, and their impacts on animal welfare are likely to expand. Hemorrhagic diseases resulting from *Culicoides*-vectored viruses, for example, can lead to millions of dollars in economic damages for producers. Chemical insecticides can reduce *Culicoides* abundance but may not suppress population numbers enough to prevent pathogen transmission. These insecticides can also cause negative effects on non-target organisms and ecosystems. RNA interference (RNAi) is a cellular regulatory mechanism that degrades mRNA and suppresses gene expression. Studies have examined the utility of this mechanism for insect pest control, and with it, have described the hurdles towards producing, optimizing, and applying these RNAi-based products. These methods hold promise for being highly specific and environmentally benign when compared to chemical insecticides and are more transient than engineering transgenic insects. Given the lack of available control options for *Culicoides*, RNAi-based products could be an option to treat large areas with minimal environmental impact. In this study, we describe the state of current *Culicoides* control methods, successes and hurdles towards using RNAi for pest control, and the necessary research required to bring an RNAi-based control method to fruition for *Culicoides* midges.

## 1. Introduction

Livestock animals are susceptible to numerous viral, protozoan, and nematode pathogens that are transmitted by *Culicoides* Latreille biting midges (Diptera: Ceratopogonidae) [1]. Disease in herds such as cattle can impact livestock producers by lowering animal weight gain, decreasing milk production, and increasing animal mortality [2]. These insects occur throughout much of the world and the effects of climate change are likely to exacerbate the risks posed by *Culicoides* [3,4,5,6,7]. Warmer temperatures are expected to increase seasonal midge activity, shorten overwintering periods, and even enhance viral replication in insects [8,9]. Effective control strategies are needed to protect susceptible herds from *Culicoides*-transmitted diseases.

Reducing pathogen transmission is aided by managing susceptible animals (e.g., stabling animals during hours with increased biting midge activity) and other cultural control methods [10]. Vector control is another aspect of a multifactorial approach needed to reduce the burden of *Culicoides* with the ultimate goal of reducing disease agent transmission [11]. However, there are few products that are labeled to target *Culicoides*. Common chemical insecticides against adult populations include pyrethroids and organophosphates [12]. These non-specific products are concerning for large-scale applications, and even insect growth regulators (IGRs), which are more specific and safer for the environment that can affect non-target insect species. Microbially derived insecticides, such as those containing *Bacillus thuringiensis* subsp. *Israelensis* (*Bti*) toxins, are touted as safer products given their biological nature and specificity (e.g., *Bti* specificity in dipteran insects) [13]. Reports suggest these products are ineffective against midges despite being highly effective in mosquitos [14]. Using RNA interference (RNAi) to suppress gene expression in *Culicoides* has only briefly been examined, and its utility as a larval control strategy is worth exploring. We summarize the impacts of *Culicoides* and recent advances in efforts to manage their populations here. We also highlight how RNAi could be used to control *Culicoides* by examining research on mosquitos as a proxy for midges. Finally, we discuss how RNAi-based insecticides in *Culicoides* could be developed and evaluated.

## 2. *Culicoides* Biting Midges

*Culicoides* are small, hematophagous flies and over 1300 species have been described throughout much of the world [1,15]. Females taking a blood meal to produce eggs can cause exceptionally irritating bites as they lacerate the skin and use a complex cocktail of salivary proteins to attenuate host responses [16]. *Culicoides* feed on many types of hosts (mammals, birds, and reptiles) depending on midge species, locality, and host availability [1]. Only a few *Culicoides* species are competent to transmit important livestock pathogens, but their behavior and ecology can vary and confound control strategies [17].

Blood-fed *Culicoides* develop eggs within a few days at optimal temperatures and oviposit them into moist substrates. The eggs rapidly hatch, and immature midges develop through four larval instars before pupating and emerging as adults. Substrate preference for oviposition depends on species. Common substrate types include manure-rich mud with a water–soil interface (e.g., *C. sonorensis* Wirth & Jones), composted material (e.g., *C. imicola* Kieffer), and animal dung pats (e.g., *C. brevitarsis* Kieffer) [17]. Yet, many larval habitats appear to be undescribed or cryptic [11,18].

The seasonality of adult midges is also dependent on species and geography. The common ecological drivers of adult *Culicoides* abundance include warm springs and autumns, higher precipitation, humidity, proximity to aquatic sources, and proximity to hosts such as cattle and sheep [19,20,21,22]. Evaluating midge abundance is accomplished primarily by trapping adult insects using carbon dioxide-baited traps, light traps, or combinations of these [23]. Recent work suggests that light-attractant trapping methods could be underestimating species richness and infection prevalence, which are important considerations in epidemiological studies [24]. Larvae may be collected from suspected habitats (e.g., manure-rich wastewater mud) and sampled directly, or adults may be sampled in emergence traps from these substrates [23]. These techniques are necessary to surveil the presence and abundance of *Culicoides*, as well as pathogen prevalence in midge populations to monitor risks to livestock.

*Culicoides* have been implicated as vectors of numerous pathogens around much of the globe. The most concerning viruses are those affecting livestock, which include bluetongue virus (BTV), epizootic hemorrhagic disease virus (EHDV), vesicular stomatitis virus (VSV), African horse sickness virus (AHSV), Akabane virus (AKV), and Schmallenberg virus (SBV) [1,17]. Oropouche virus (OROV) is one of the few *Culicoides*-transmitted pathogens that can cause disease in humans. Other pathogens include lumpy skin disease virus (LSDV), bovine ephemeral fever virus (BEFV), and Simbu serogroup virus, which are concerning for livestock [25,26]. Clinical presentations in most livestock-associated diseases include fever, lesions, edema, and lameness. Disease can lead to mortality in susceptible animals, such as BTV in sheep, EHDV in white-tailed deer, and AHSV in horses [1]. Beyond the loss of livestock, further considerations for *Culicoides*-associated diseases include reduced animal weight, lowered milk production, and fetal abortion. These outcomes have significant economic costs for livestock producers. The economic impact of BTV-8 in France and the Netherlands in 2007 alone was over USD 1.4 billion, with larger impact estimates at over USD 3 billion globally [27]. Livestock producers are under immense pressure from *Culicoides*-associated diseases, which emphasizes the need for effective population management methods.

Modeling shows strong associations between temperature and *Culicoides* seasonal abundance, as evidenced by recent studies [19,28,29,30]. Warming climates will likely lead to range expansion for some *Culicoides* species, even in regions where temperatures become too warm for established species, and others that are better adapted to these conditions may become established [9,31]. These midges will continue to play a major role in animal welfare and a diverse set of control options are necessary to combat these pests.

## 3. Current Control Strategies

Most biting midge species are nuisance pests and a few species vectors are livestock-associated pathogens. As such, most control strategies currently employed for midge control focus on reducing midge abundance in animal agriculture systems. The current strategies can broadly be grouped into cultural control, chemical insecticides, and microbial insecticides. These have been extensively reviewed previously [10,32,33,34]. In this study, we will describe updates to the field and their implications for the state of *Culicoides* control.

### 3.1. Cultural Control

Vaccinating animals can reduce viral transmission and disease severity. Inactivated vaccines are effective, but some can be expensive to produce, whereas modified live virus vaccines pose the risk of reassortment with wild-type viruses [4,35]. This issue is also complicated in regions where multiple viral serotypes are present and vaccinations lack the appropriate coverage. Herd management may play a role in amplifying viral spread. A study in India found buffalos, which are asymptomatic when infected with BTV and were placed as a buffer to susceptible sheep herds, were instead serving as reservoirs for the virus and leading to disease outbreaks [36]. This finding suggests that effective vaccine campaigns will need to consider local virus reservoirs. The authors of another recent study discuss considerations for not vaccinating herds, given the BTV vaccine’s prohibitive costs and loss of protective immunity in herd animals [37].

Predictive measures such as disease and vector modeling are increasingly being used by examining past outbreaks to predict future outbreaks [38,39,40,41,42]. These models are informed by basic *Culicoides* collection efforts globally, which link local ecology to species richness and distribution as well as how weather patterns affect midge abundance [21,43,44]. One study detected *Culicoides* in a region previously thought to have been inhospitable in high-altitude regions of China [45]. These data contribute to advances in large, regional modeling efforts that can influence herd movement and vaccination campaigns in a cost-effective manner.

Landscape alteration for *Culicoides* management could potentially reduce midge abundance. In livestock rearing operations, removing manure and other organic waste restricts habitable larval development sites. A study from 2014 evaluated the impact of removing a wastewater collection site from a California dairy farm and compared *C. sonorensis* abundance to a control site [46]. However, the authors found an opposite effect where more *Culicoides* were collected from the farm lacking a wastewater pond. These findings suggest that *C. sonorensis* is likely using other agricultural-associated water sources. Efficiently identifying these sites to potentially remove them or treat them may reduce these spillover events [47]. Another consideration for landscape alteration is separating livestock animals from wild animals that harbor viruses, such as BTV and EHDV in cervids [48,49,50,51]. The analysis of midge bloodmeals can better inform livestock producers which wild animals are near managed herds and could inform measures taken to reduce their contact with midges feeding on both types of animals.

Perhaps the most problematic factor contributing to effective *Culicoides* control is the identification of larval habitats. This is substantiated in a recent article where Braverman et al. [18] describe the collection of 58 adult *Culicoides* species in suctions traps, but larvae of only 15 species were collected from substrates in the same region of Israel. Their work went on to assess multiple types of substrates, from organic-poor sandy soil to organic-rich manure-infused mud, to tree holes and water pools in pockets of vegetation in cropland. The authors found *Culicoides* in all types of substrates with different species preferring different environments. Two articles assessed midge oviposition preference in Florida by using combined field and laboratory experiments that highlight the importance of understanding larval development site choice for important vector species [52,53]. Wong et al. [54] found that *Culicoides* in Southern California preferred ovipositing onto moist soil within 5 cm of the water line in agricultural wastewater ponds. The authors suggest altering the pond water levels to desiccate freshly deposited eggs from the previous night. Measures such as these are relatively simple and may be effective at controlling those species that utilize large water sources as larval habitats. More basic research is needed to identify cryptic breeding habitats and will inform larval control measures.

### 3.2. Chemical Insecticides

The most common methods of controlling *Culicoides* have traditionally been through chemical applications in the form of adulticides and repellents on animals and structures, and larvicides in known or suspected habitats suitable for immature development [32]. The use of insecticides poses a risk for fostering insecticide resistance in *Culicoides* as it has with other pest insects [33]. While specific insecticide resistance does not appear to be an imminent threat, laboratory studies have shown variable efficacy of insecticides between *Culicoides* species and within species’ populations [10,12,32]. This observation promotes the need for new control measures, including those beyond chemical insecticides.

Topical insecticide application to host animals can create targeted sources of exposure for adult midges. However, both insecticides and repellents are quick to wash off in rain and they will naturally degrade under average environmental conditions in the field if they are not reapplied often [32]. In a recent study using ivermectin and deltamethrin applied topically to the midline of cattle, the authors found no significant differences in the number of blood-fed females collected from treated and control animals [55]. The authors note that the midges appeared to have been exposed to lethal levels of insecticide. Although it is a potential route of adult control, these methods would not initially prevent bites from potentially infected females.

Effective midge population control likely requires employing multiple methods. One recent study used deltamethrin to control adult insects and both *Bti* and diflubenzuron to treat larval habitats [56]. This study found reduced numbers of *Culicoides* on working sheep farms that were treated with all three insecticides compared to untreated farms that served as controls for the experiment. Larval treatment areas were selected for their composition of moist habitats with organic matter. Although it was successful, this approach may not always be feasible when the breeding habitats for the species of concern are unknown.

Essential oils are thought to provide a more natural alternative for repelling or killing adult *Culicoides*, but their efficacy has mixed results [57]. Benelli and colleagues [33] used an inexpensive neem product to treat livestock waste runoff ponds. This treatment successfully discouraged adult oviposition onto the mud substrate compared to controls. The researchers examined adult emergence as a metric but did not quantify the larval populations around the treated area. The neem cake used was noted to be less expensive than the concentrated active ingredients that are produced, namely azadirachtin. The adult repellency distance for this product is unknown, so it is unclear how much area needs to be treated and how frequently. No single chemical insecticide or repellent has been identified as an efficient and effective treatment for controlling *Culicoides*. Combination approaches to manage adult and larval stages would be the most effective so long as insecticide resistance and aquatic ecosystem health are monitored closely [58].

Treating larval stages of *Culicoides* in semi-aquatic ecosystems is complicated by dilution effects, poor mixing of hydrophobic compounds with water, and environmental degradation. In addition, midges rarely occupy these habitats on their own, and the effects of chemical insecticides on non-target organisms is an important consideration. Studies have found pesticides such as neonicotinoids and fipronil reduced micro- and macro-invertebrate abundance in habitats near agricultural operations [59]. Cypermethrin was detected in soil samples more than three months after treatment, and many trophic levels were found to have been negatively affected [60]. Semi-natural experiments evaluated permethrin toxicity in containers containing multiple trophic levels of aquatic animals [61]. Here, the authors showed that aquatic insects were highly susceptible to the insecticide, but it should be noted that these systems were stagnant, and the level of insecticide used was higher than the recommended treatment rate. Concerns for off-target environmental effects from chemical insecticides lie not only with the agricultural producers but also with household consumers who may be unaware of insecticide toxicity to non-target species.

### 3.3. Microbial Insecticides 

Entomopathogenic fungi and bacterial-derived insecticides are commonly used in mosquito control as bio-rational alternatives to traditional chemical insecticides. Entomopathogenic fungi appear to kill *Culicoides* and do not have off-target effects on those non-pest insects tested in one recent study [62]. Distributing fungal spores into the environment at a large scale should be considered carefully but use in livestock housing units may be an effective strategy to infect midges upon contact. While this scenario would not prevent bites, it would control adult populations and kill midges before they can oviposit as mortality was observed in ~24 h.

Perhaps the most well-known are those products containing insecticidal toxins from *Bti*, which are toxic to dipteran insects such as mosquitos and black flies [13]. Using *Bti* is quite common in large-scale applications and in many types of consumer products for mosquito control. However, microbial *Bti* and commercial formulations of *Bti* appear to have limited efficacy as larvicides against midges. A recent study determined the lethal concentrations of *Bti* in *Culicoides* larvae that were collected in Sardinia and found the values were much higher than those seen in most mosquito species [14]. The authors show *Bti* appeared to work best compared to two other insecticides derived from the bacteria *B. sphaericus* and *B. laterosporus*. Two North American biting midges, one *Culicoides* sp. and one *Leptoconops* sp. Skuse, were also unaffected by *Bti* products in one early study [63]. The refractory nature of midges to *Bti* toxicity has been linked to an acidic gut environment, which is not permissible for toxin activation [64]. The alternative formulations of *Bti* insecticides could alter the larval gut pH, but current *Bti* products appear to be unfavorable for *Culicoides* control. The environmental impacts of biological control agents are expected to be less than those of traditional chemical insecticides, but the risks are not negligible and should be included in any risk assessment.

## 4. RNAi for Insect Control

RNA interference (RNAi) is a conserved and highly specific mechanism for suppressing target genes. For use in insect pest control, RNAi causes mortality or other favorable outcomes as a result of suppressing physiologically important proteins and enzymes. RNAi-based insecticides use nucleic acids (e.g., double-stranded RNA and dsRNA) complementary to the genetic sequence of target genes in the pest to suppress target transcript levels. Thus, the effects should be highly specific to a target insect, and RNA-based treatments should be environmentally labile. However, while promising, there are numerous hurdles to overcome in developing efficient RNAi-based treatment options in insects.

### 4.1. Mechanism and Function

**Figure 1:**
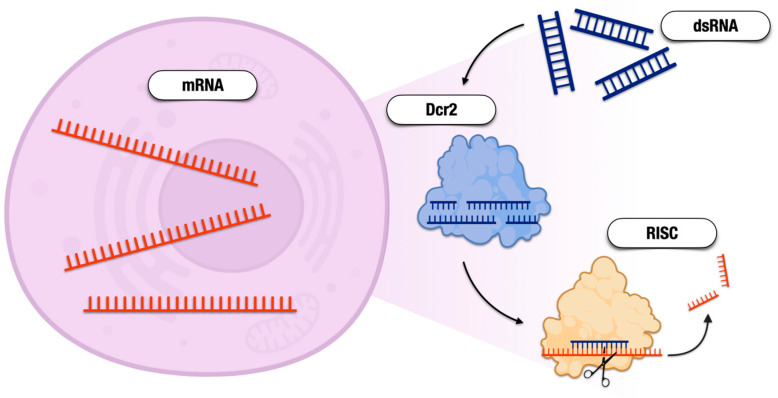
Representation of the siRNA pathway. Double-stranded RNA (dsRNA) is internalized in the cell where it is cleaved into siRNA by Dicer-2 (Dcr2). One strand of siRNA serves as a guide for mRNA in the RNA-induced silencing complex (RISC). Argonaute-2 in the RISC degrades mRNA and renders it unusable, leading the gene suppression.

The term RNAi encompasses multiple overlapping pathways wherein RNA molecules are degraded. The major pathways are reviewed by Zhu and Palli [65] and discussed briefly here. The small interfering RNA (siRNA) pathway begins when dsRNA is bound by accessory proteins, R2D2 or Loquacious, and is enzymatically cleaved by Dicer-2 (Dcr2). These cleavages result in small dsRNA products of approximately 22 bp and are termed siRNA molecules. Each siRNA molecule is incorporated into a complex of accessory proteins that include the enzyme Argonaute-2 (Ago2). One strand of RNA, termed the passenger strand, is degraded while the remaining strand serves as a guide to bind complementary mRNA molecules. This complex of proteins and RNA is referred to as the RNA-induced silencing complex (RISC). The siRNA pathway can use exogenous or endogenous dsRNA as a starting substrate and has roles in cleaving host mRNA to silence gene expression, cleaving viral RNA products, and silencing transposons (Figure 1).

There are two other pathways that overlap in functionality with the siRNA pathway and whose endpoints are the degradation of RNA. The microRNA (miRNA) pathway causes host gene silencing from host-derived RNA templates. The miRNA pathway resembles the siRNA pathway albeit with a different set of proteins (e.g., Dcr1, Ago1). The final pathway, piwi-interacting RNA (piRNA), has a role in degrading host transposon transcripts. These pathways are conserved in insects, and some proteins have undergone gene duplication in some species [66]. Insecticidal RNAi products are expected to be processed by the siRNA pathway where exogenous dsRNA molecules are taken up by cells, processed as above, leading to gene suppression, though the other two pathways can also play small roles in this function [67].

### 4.2. Insecticidal Properties

Any number of physiologically important genes could be targets for RNAi-based suppression. Ideal RNAi targets are genes that are highly expressed with large mRNA pools, sensitive to RNAi, and encode proteins with short half-lives [68]. If RNAi is only a transient response in an insect, long-lived proteins may not be affected by dsRNA treatment. A screen of candidate RNAi targets in *Anopheles gambiae* Giles found suppressing actin, a leukocyte receptor complex member, and offtrack genes to be insecticidal in larvae fed on yeast, producing short-hairpin RNAs for these targets [69]. This study also showed that the treatment led to up to 100% mortality in laboratory trials. Chitin synthase genes are also common targets for RNAi in insects. Chitin synthase 1 (*CHS-1*) is involved with cuticular chitin synthesis, whereas chitin synthase 2 (*CHS-2*) synthesizes the chitin of the peritrophic matrix [70]. Suppressing these genes in mosquitos can lead to decreased chitin formation and greater susceptibility to insecticides, such as diflubenzuron [71,72]. Other targets in six mosquito species are reviewed by Munawar et al. [73]. Target selection is the first of many important steps towards developing RNAi-based insecticides, and there are many considerations for optimizing their delivery.

### 4.3. Barriers to Implementation

**Figure 2:**
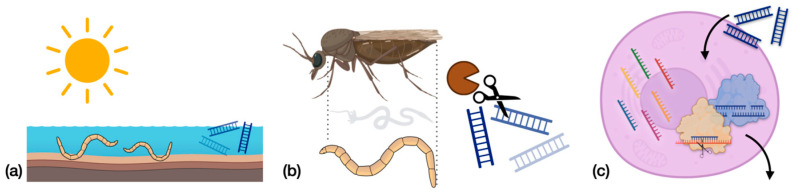
Examples of barriers towards effective RNAi treatments in insects: (**a**) Environmental degradation and dilution effects; (**b**) enzymatic degradation of dsRNA by enzymes (dsRNases) in hemolymph and gut fluid; (**c**) poor dsRNA uptake, deficient core RNAi-associated proteins and enzymes, refractory target genes, and poor systemic spread in cells (adapted from [75]).

Creating efficient RNAi-based insecticides requires understanding the properties of the specific insect pest. For example, beetles are usually highly susceptible to dsRNA treatment, and target suppression of greater than 90% can be achieved with low doses of dsRNA. In contrast, moths and butterflies are quite refractory to treatment [74,75]. Numerous factors may contribute to these discrepancies and include the stability of dsRNA molecules, uptake and processing of dsRNA in the insect cells, expression of core RNAi machinery, and viral suppressors of RNAi, among others [75]. Currently, very little is known about how any of these potential barriers may affect RNAi efficiency in *Culicoides* midges, but these are important factors in developing RNAi as a management tool for these livestock pests (Figure 2).

One of these barriers is the expression of enzymes which degrade dsRNA molecules, leading to low RNAi responses in insects. These enzymes are termed dsRNA-degrading enzymes or nucleases (dsRNases), and their abundance, diversity, tissue-specific expression, and activity are investigated in many insect orders [76,77,78,79]. Suppressing these nucleases by RNAi to enhance RNAi has been investigated in *Aedes aegypti* Linnaeus [80] and in a tephritid fruit fly [81] with success. Specifically, Giesbrecht et al. [80] showed midgut specificity for two larval mosquito nucleases that contributed to the degradation of orally delivered dsRNA. Identifying and overcoming these nucleases may be essential for the oral delivery of dsRNA-based control agents.

## 5. Strategies to Enhance RNAi 

Overcoming barriers to dsRNA production, application, environmental stability, and uptake are avenues of ongoing investigation. Specific strategies will likely need to be developed for different *Culicoides* species given their diversity of habitats and behaviors. There are many methods available for creating more stable RNAi products and producing RNAi products at scale.

### 5.1. Nanoparticles

Free dsRNAs in the insect gut lumen and hemolymph are exposed to enzymes that may degrade these molecules before they can enter cells and initiate an RNAi response. Nanoparticles made of organic or inorganic and natural or synthetic materials can bind dsRNA molecules to reduce enzyme access to these molecules [82,83]. Chitosan is a common material for constructing these nanoparticles. Here, electrostatic interactions of the negatively charged dsRNAs and the positively charged amino groups on the chitosan polymer lead to the formation of nanoparticles [84]. These nanoparticles can be fed to larval insects in a food source. One study showed two chitin synthase genes in *An. gambiae* were suppressed in larvae fed 5 μg of dsRNA-chitosan nanoparticles in food each day for 4 days [71]. The transcripts of both *CHS-1* and *-2* were suppressed by 50–60% in their respective treatments, led to noticeable phenotypes in larvae, and increased larval mortality. Similarly, Das et al. [85] compared the activities of chitosan nanoparticles to those of carbon quantum dots and silica nanoparticles. The authors showed significant suppression of two genes when dsRNA was bound to chitosan nanoparticles and one gene when dsRNA was bound to carbon quantum dot nanoparticles. Additionally, these nanoparticles were shown to have some insecticidal properties on their own (i.e., when bound to ds*GFP*). It is noted that these materials are biodegradable and have low toxicity, which could ease worries about their use in the environment [85]. Developing nanoparticles and carrier compounds with selective charges is hypothesized to create specificity for delivering dsRNA to tissue types in insects [86]. These advances are promising routes towards improving dsRNA stability and delivery of insecticidal treatments.

### 5.2. RNA Structures

Designing dsRNA molecules with sequences of complementary bases separated by a segment of non-complementary bases encourages loop formation. A common example is short hairpin RNA (shRNA) molecules with a single loop at one end of the target sequence formed by the non-complementary bases. These are used extensively in mosquito studies and can be synthesized in vitro or expressed in microbial systems [80,87,88]. These molecules are effective for target gene suppression and have been shown to reduce arboviral replication via RNAi suppression in cells and animal models, which can enhance antiviral activity in insects and reduce viral spread in vector species [89,90].

As an alternative to shRNAs, Abbasi et al. [87] constructed RNA molecules containing closed ends at both termini and termed these paperclip RNA (pcRNA). The authors show these pcRNA (23 bp) molecules to be as efficient as long dsRNA (200 bp) molecules, as well as siRNA and shRNA (23 bp) molecules. Importantly, they also show that pcRNA is taken up by cells via a novel mechanism that is different from that of shRNAs and long dsRNAs. This finding is particularly significant because one documented mechanism of resistance to RNAi is via the inhibition of the uptake of long dsRNAs. In *Diabrotica virgifera virgifera* LeConte (Coleoptera: Chrysomelidae), beetles exposed to dsRNA targeting *Snf7* became resistant to dsRNA treatment after repeated exposures because reduced dsRNA uptake, not dsRNA degradation, in the gut lumen resulted in lowered RNAi efficacy [91]. The implications of this work will need to be explored in greater detail as RNAi-based insect control products are developed. However, secondary RNA structures may create more resilient dsRNA molecules with alternative uptake in insect cells.

### 5.3. Bacterial Expression

Expressing dsRNA in microorganisms provides an alternative for mass in vitro dsRNA production. A common approach uses the L4440 plasmid that contains inverted T7 promotors to create an expression template for target dsRNA. Once the plasmid is constructed, it can be transformed into bacteria, such as the HT115 (DE3) *Escherichia coli* strain which lacks *RNaseIII,* preventing dsRNA product degradation. Synthesis is then induced with isopropyl β-d-1- thiogalactopyranoside (IPTG). Ahn et al. [92] provide an overview of this process and compare heat and sonication methods for liberating dsRNA from *E. coli* after production. In this study, the authors found that dsRNA yields from a 25 mL culture volume were estimated at 488 μg, 238 μg, and 97 μg for sonication, heat, and conventional harvest methods, respectively. In a different study, *E. coli* was induced with lactose instead of IPTG and lysed with chlorhexidine to liberate dsRNA, which the authors describe as inexpensive alternatives to those previously described [72]. In this work, *Ae. aegypti* larvae showed a 50% suppression of chitin synthase A when treated with 400 ng of dsRNA in their rearing water, and this was associated with 50% mortality in 4th instar larvae after four days. Direct application of dsRNA-expressing *E. coli* into rearing water, or their incorporation into a food source, may also assist with the delivery and protection of dsRNA in the insect gut. Taracena et al. [93] show that this methodology can also be used to feed adult mosquitos through a sugar-*E. coli* meal to achieve significant target knockdown. A recent study also described the production of minicells from *E. coli* producing dsRNA, which resulted in protective encapsulations of dsRNA for fungal control [94]. This technology appears to be adaptable for diverse pests and is worth further investigation for use in *Culicoides*.

Researchers are also exploring the utility of engineering symbiotic bacteria to express dsRNA. One study used a symbiont expressing a dsRNA hairpin targeting *Rhodnius prolixus* Stål, a vector of Chagas disease, and the authors found a significant reduction in target mRNA levels and reduced egg production in adult females [95]. Another study evaluated symbiote expression in honey bees and their associated gut bacterium, *Snodgrassella alvi*, which produced dsRNA against *Varroa destructor* Anderson and Trueman mites [96]. The symbiont successfully colonized the bee guts and significantly reduced feeding mite survival. Symbiotic bacteria may be an ideal delivery system given their close association with target species and certain tissue tropisms in the host, but delivery methods for delivering the engineered symbiotes to their hosts and having these spread from host to host need further development.

### 5.4. Fungal Expression 

Perhaps one of the most promising methods for mass-producing dsRNA in a microbial system uses baker’s yeast, *Saccharomyces cerevisiae* [88]. This organism is unique for its production scalability and stability when dried. When compared to heat-killed bacteria expressing dsRNA, *S. cerevisiae* performed just as effectively (greater than 75% mortality) in larval mosquito bioassays [69]. The same research group later showed high efficacy of yeast-based larvicides expressing shRNA targeting an olfactory receptor neuron (*sema1a*) against *Ae. aegypti*, *Ae. albopictus* Skuse, *An. gambiae*, and *Culex quinquefasciatus* Say with mortality rates greater than 80% [97]. This group has gone on to develop multiple yeast-based insecticidal treatments targeting mosquito physiology [98,99,100]. Yeasts provide some increased stability as they can be readily dried and reconstituted in mosquito larval environments [88].

### 5.5. Viruses

Viruses present a unique delivery option for dsRNA as they can be engineered to deliver dsRNA-expressing nucleic acid constructs and virus-like particles can be used as empty vessels to deliver dsRNA cargoes into cells [101]. Gu and colleagues [102] created recombinant mosquito densoviruses containing shRNA constructs targeting either *Ae. aegypti* or *Ae. albopictus V-ATPase*. Their results show a broad viral affinity for multiple mosquito tissues, target suppression in cells and larvae, and an increase in larval mortality after exposure to the virus via soaking. In addition, plant viroids provide a unique scaffold for dsRNA production and have been shown to be insecticidal in *D. virgifera* [103]. Virus host specificity, genome type, tissue tropism, infectivity, and persistence are among the considerations that should be investigated as these avenues are developed for RNAi pest control in biting midges.

### 5.6. Algal Expression

Aquatic insects, or those with aquatic life stages, are likely to encounter algae in natural ecosystems. These organisms have been investigated as dsRNA production systems for mosquito control in several studies. One group engineered *Chlamydomonas reinhardtii* chloroplasts to produce dsRNA targeting *3-HKT* in *An. stephensi* Liston [104]. The authors report significant target suppression and increases in larval mortality when larvae were fed transgenic algae. Another study used the same microalgae to express dsRNA against *HR3* in *Ae. aegypti*, and similarly found target suppression and increased larval mortality compared to controls [105]. Other studies have used both *Chlamydomonas* and *Chlorella*, which includes strains harvested from water sources near the associated research institutions to kill *Ae. aegypti* and *Ae. albopictus* larvae [106,107]. These studies are promising because they have shown successful transformation of both laboratory and natural algal strains. In addition, Fei et al. [107] found their transformed *Chlorella* sp. to be larvicidal in semi-field trials. While these studies are promising, transforming other algal species and strains that are specific to a pest’s natural environment may not be as easy. There are a number of considerations for the successful engineering of these algae, reviewed here [108].

## 6. Outlook for Controlling *Culicoides* Using RNAi

The first publication to examine RNAi in *Culicoides* used cultured cells to show BTV (a dsRNA virus) was likely degraded by Dcr2 and resulted in 21 nt virus-derived small interfering RNA (viRNA) molecules [109]. Mills et al. [110] described experiments to knock down the transcript levels of a target gene in adult *C. sonorensis* by RNAi. The authors showed that the core RNAi machinery is present in *C. sonorensis* (i.e., *Ago2*, *Dcr2*, and *R2D2*) and that intrathoracic injection of dsRNA led to approximately 45% transcript suppression after 5 days. These results show that *C. sonorensis* is sensitive to dsRNA, at least by injection, and that RNAi pathways are functional.

Translating these findings into treatment options for controlling *Culicoides* requires more foundational work, however. Approaches reviewed by Silver et al. [82] and in this study include naked dsRNA treatments, complexing dsRNA to nanoparticles, using viral vectors, and expressing dsRNA in microbial systems. These treatments could be applied to potential or known *Culicoides* larval habitats to control larval midges. *Culicoides* are generalist feeders and therefore are expected to consume bacteria, fungi, and algae. Hydrogel baits for larval mosquitos have been developed to hold phagostimulants and insecticides [111]. However, these are formulated to float on the surface of water. Larval *Culicoides* inhabit just the first few centimeters of substrate to facilitate respiration. Ideally, treatments would be spread into these systems and incorporated into the shallow substrates of ponds, muddy depressions, compost, and manure. Alternatively, sugar baits or similar attractants containing dsRNA could be used to target adults [10,93].

Desired outcomes for RNAi-based control methods include mortality and altering pathogen transmission. In larval *Culicoides*, these could target physiologically important genes that have been explored in mosquito literature. For example, suppressing chitin synthases can affect cuticle formation during molting between larval instars and during pupation and reduce peritrophic matrix formation in the larval gut. Combinatorial treatments may prove useful in increasing efficacy. Bacteria and other disruptors of peritrophic matrix integrity could degrade *Culicoides* gut integrity, and chemical chitin synthase inhibitors such as diflubenzuron can alter molting efficiency [71,112]. One interesting target is the sex selection pathway. In mosquitos, studies have targeted genes resulting in altered male:female ratios, which show promise for reducing female populations (i.e., the blood-feeding stage) [113,114]. Other targets may influence pathogen fitness in the host, insect fecundity, and insect host-seeking ability.

Perhaps the most important aspect of RNAi-based insect control is its high specificity and low off-target effects when compared to traditional insecticides. The regulatory framework for reviewing dsRNA products in the United States and in the European Union is reviewed by Dietz-Pfeilstetter et al. [115]. These authors, and others, encourage toxicity studies on non-target organisms to ensure specificity [116]. One study also examined oral toxicity in a mammalian system, which found no negative outcomes [117]. These considerations are not only important for the scientific community but also for public perception. One study engaged community members in a potential treatment site where yeast-based dsRNA insecticides were planned to be used [118]. The authors found that the community was well-versed in mosquito biology and open to new insecticides if they were safe. Next-generation approaches to control *Culicoides* have been proposed to include sterile insect technique, *Wolbachia*-based sterility, and transgenic alterations [119]. Many of these techniques have been untested in large systems and could draw public criticism. RNA interference approaches may be more tolerated by the community as they do not affect insects’ genomes and have transient properties. However, the release of any transgenic organism, including those discussed here (i.e., bacteria, fungi, and algae), will require careful evaluation and regulatory approvals, especially in natural areas.

## 7. Conclusions

**Figure 3:**
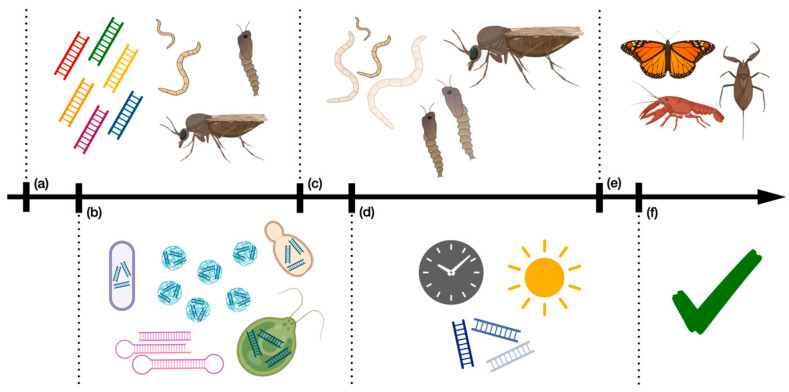
Example development pipeline for RNAi-based insecticides against *Culicoides*: (**a**) Selecting target gene(s) and insect developmental stage; (**b**) developing RNAi enhancers (e.g., microbial expression); (**c**) evaluating treatment efficacy (e.g., mortality, molting efficiency, etc.); (**d**) optimizing concentration, reapplication rates, and environmental stability; (**e**) determining effects on non-target organisms; (**f**) seeking regulatory approval.

Effective *Culicoides* control by cultural methods and chemical and biorational insecticides has been challenging for these insects. RNAi-based insecticides are an exciting avenue for advancement in the field but are not without hurdles towards widespread implementation. Effective control will require finding ideal genes for suppression in target midge species, optimizing delivery methods, designing application methods that are efficient and stable in the environment, monitoring the effects on target and non-target species, and working with regulatory bodies and the public for approvals and acceptance (Figure 3). Many foundational studies on RNAi in *Culicoides* remain to be examined, but this field has great potential as such products could drastically reduce the need for broad-spectrum insecticides that have detrimental effects on non-target organisms and the environment. Given the current lack of effective control strategies, *Culicoides* are an important target for advanced insecticide development.

## Data Availability

No new data were created or analyzed in this study. Data sharing is not applicable to this article.
